# Health State Utilities Associated With Treatment Burden in Cystic Fibrosis: A Patient Valuation Study

**DOI:** 10.1016/j.chpulm.2024.100097

**Published:** 2024-08-23

**Authors:** Rory A. Cameron, Jessie Matthews, Daniel Office, Mark Rowley, Janice Abbott, Nicholas J. Simmonds, Jennifer A. Whitty, Siobhán B. Carr

**Affiliations:** a Norwich Medical School, University of East Anglia, Norwich, England; b National Institute for Health Research, Applied Research Collaboration East of England, Cambridge, England; c Adult Cystic Fibrosis Centre, Royal Brompton Hospital, London, England; d patient representative, London, England; e School of Psychology, University of Central Lancashire, Preston, England; f National Heart and Lung Institute, Imperial College, London, England; g Department of Paediatric Respiratory Medicine, Royal Brompton Hospital, London, England; h Evidera Inc, London, England

**Keywords:** cystic fibrosis, health-related quality of life, health state utility, patient and public involvement, time trade-off, treatment burden

## Abstract

**Background:**

Although recent advancements in the treatment of cystic fibrosis (CF) have improved survival, reducing high levels of treatment burden remains a priority issue for many people with cystic fibrosis (pwCF). However, economic evaluations of novel interventions may fail to capture their impact on treatment burden due to a lack of suitable outcome measures. This study aimed to estimate health state utilities (HSUs) for changes in treatment burden associated with different CF treatments.

**Research Question:**

What value do pwCF place on changes in treatment burden associated with IV antibiotic treatment of pulmonary exacerbations, use of inhaled medicines, and physiotherapy?

**Study Design and Methods:**

Adults attending a specialist CF center were invited to participate in a web-based time trade-off interview. Participants valued their own health and five health state vignettes describing varying levels of intensity of physiotherapy, use of inhaled medicines, and IV antibiotic treatment. HSUs for additional instances of each treatment type were estimated using mixed effect linear regression models.

**Results:**

Fifty one pwCF completed the interview (median age, 30 years; range, 19-66); 53% were female; mean FEV_1_ % predicted was 65% (SD, 20%). Mean utility scores for own health were very similar between the EQ-5D index value (0.81; SD, 0.20) and the time trade-off value (0.82; SD, 0.20); however, limited concordance was observed at the individual level. Adjusted utility decrements associated with treatment burden were −0.037 (SE, 0.008) for an additional annual IV antibiotic treatment, −0.029 (SE, 0.014) for an additional daily physiotherapy session, and −0.019 (SE, 0.013) for an additional daily inhaled medicine.

**Interpretation:**

In this study, increasing treatment burden was associated with decreasing HSU values. The utility decrements associated with treatment burden changes suggest meaningful differences in health-related quality of life for pwCF. These findings align with existing literature on the impact of treatment burden on health-related quality of life, and highlight the importance of considering treatment burden in economic evaluations of interventions in CF.

**Take-home Points**

**Study Question:** What value do people with cystic fibrosis (CF) place on changes in treatment burden?

**Results:** In this study, statistically significant utility decrements within the range of commonly reported minimally important differences were associated with increased IV antibiotic and physiotherapy treatment occasions, whereas nominal utility decrements were associated with increased inhaled medicine treatment occasions.

**Interpretation:** Results suggest that relatively modest changes in treatment burden represent meaningful differences in health-related quality of life for people with CF and highlight the importance of considering treatment burden in future evaluations of CF interventions.

Sequential improvements in the treatments available for cystic fibrosis (CF) have led to markedly improved survival, but people with cystic fibrosis (pwCF) still experience debilitating symptoms, reduced life expectancy and health-related quality of life (HRQoL), and a high level of perceived treatment burden (defined as the health care workload they experience).^1^ Although there is emerging evidence that the introduction of highly effective modulator therapies (HEMTs) has reduced the incidence of pulmonary exacerbations (PExs)^2^ and levels of perceived treatment burden,^3^ these treatments have not eliminated the need for complex and intensive treatment regimens. Furthermore, approximately 10% of the CF population are not eligible for HEMT.^4^ The 2022 James Lind Alliance revisitation of research priorities in CF found that simplifying treatment burden remains a top 10 objective in the CF community.^5^

Although the primary objectives of treatment in CF focus on preventing progression of lung disease and managing nutrition and CF-related complications,^6^ novel interventions may have tangential beneficial effects, including on perceived or objective treatment burden levels. Research has explored the potential to reduce treatment burden through innovations in drug delivery,^7^ self-management interventions,^8^ and treatment rationalization.^9^ Our ability to incorporate treatment burden-related benefits of such interventions in health economic evaluations however is hampered by a lack of suitable outcome measures. Commonly used generic HRQoL instruments (eg, EQ-5D) do not measure treatment burden, and although two options to derive utility scores from the disease-specific Cystic Fibrosis Questionnaire Revised (CFQ-R) measure are now available,^10^^,^^11^ neither uses the treatment burden domain of that instrument. Consequently, economic evaluations may undervalue the benefit of interventions that have a positive impact on treatment burden.

Health states measured by HRQoL instruments are valued using stated preference methods; this is most commonly the time-trade-off (TTO), but other methods including the standard gamble and the discrete choice experiment are also widely used.^12^ Each is underpinned by a different theoretical foundation but in essence all result in a relative weighting of the health states described by the HRQoL instrument. Known as health state utilities (HSUs), these weights are derived from surveys of a population’s relative preference for each health state.^12^ The utility value of a health state, together with the time spent in that health state, may then be used to estimate quality-adjusted life years as the primary outcome measure of an economic evaluation, used in health technology appraisal processes.^12^

There is ongoing debate based primarily on normative arguments regarding the most appropriate population (patients or general public) for eliciting utility values^13^; however, many health technology assessment agencies (Institute for Clinical and Economic Review in the United States, National Institute for Health and Care Excellence in the United Kingdom, Canadian Agency for Drugs and Technologies in Health in Canada) require general population values to be used wherever possible.^13^ A key argument supporting patient valuations is that patients understand the HRQoL impact of their condition better than someone trying to imagine it, particularly where uncommon treatment processes are involved (eg, those considered in this study).^12^

This study aimed to estimate HSU values for changes in level of treatment burden associated with IV antibiotic treatment of PEx, inhaled medicines, and physiotherapy.

## Study Design and Methods

This research was part of VALU-CF, a cross-sectional study concerning the measurement and valuation of CF-specific HRQoL.^14^ It uses a vignette-based TTO interview to elicit utility values for specific health states from pwCF. The TTO method is a choice-based approach for eliciting preferences for alternative health states.^15^ Respondents are asked to state their preference for living for a fixed number of years in a health state of interest and a variable number of years in a comparator health state (usually full or perfect health). The period in the comparator state is varied iteratively until the point at which the respondent is indifferent between *x* years in full health and *y* years in the health state of interest (whereby equivalence is assumed). This point of indifference is used to estimate the utility value for that health state. The vignette-based TTO approach is recognized as a convenient and appropriate tool for the isolation and measurement of the utility impact of specific attributes of a disease or treatment processes.^16^

The VALU-CF study was approved by the NHS Health Research Authority (REC 19/YH/0423). All participants provided informed consent to participate and for linkage of their UK CF registry data to the study.

### Participants

Patients who had previously completed one of two surveys within the VALU-CF study^17^^,^^18^ and had consented to further follow-up were invited to participate in the TTO interviews. We sequentially recruited patients after routine quarterly clinic visits to a target sample of 50, mindful that pwCF ineligible for HEMT account for only 10% of the UK CF population; however,^4^ we purposefully recruited a larger proportion of the sample from this group to ensure that we were able to adequately capture their preferences. Fifty-two respondents (including 16 not receiving HEMT) were recruited. Inclusion criteria required the participants to have a CF diagnosis, be > 18 years of age, and have the mental capacity to complete the interviews. In-patients experiencing acute PEx and judged by the research nurse to be too unwell to be approached were excluded. No further inclusion or exclusion criteria were applied to the TTO study.

A £25 online voucher was offered as an incentive to participate. Participant information and informed consent was provided before starting the interview.

### Health States

The health states vignettes were based on a focus group consultation with clinical and patient experts,^17^ which identified physiotherapy, nebulizer use, and IV antibiotics as the most burdensome elements of CF treatment. The precision of the HSU values estimated is a function of both sample size and number of vignettes valued for each treatment burden aspect. A pragmatic decision was made to prioritize valuation of HSU associated with IV antibiotic use (two vignettes) over physiotherapy and nebulizer use (one vignette each). Good practice guidance was followed when constructing the descriptive vignettes.^12^

**Figure 1:**
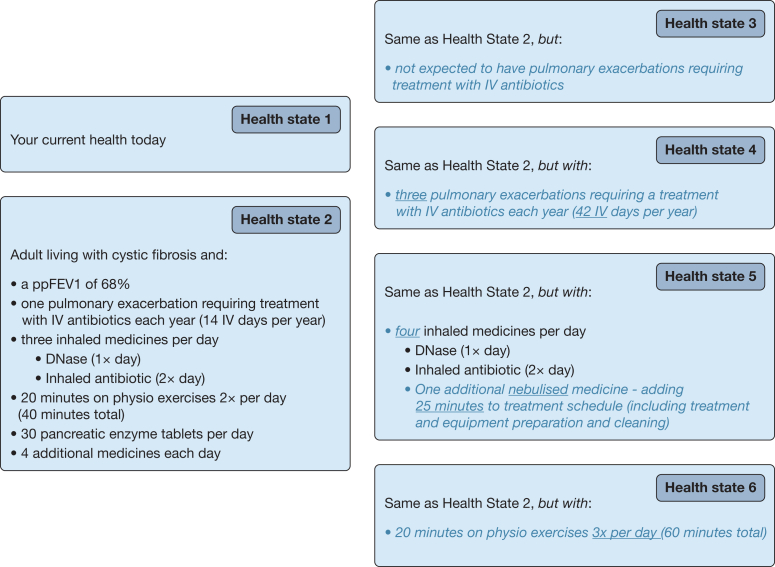
Health state vignettes shown to participants during the time trade-off tasks. Health states 3 through 6 were shown in the same format as health state 2, with changes highlighted in blue text. DNase = Deoxyribonuclease; ppFEV1= FEV_1_ % predicted.

Participants valued five structured format health state vignettes (Fig 1). They also valued their own (current) health (health state 1) to facilitate a comparison of the performance of the TTO, relative to the EQ-5D-5L measure. To maximize relatability among participants, the base case CF health state (health state 2) aimed to reflect the average person with CF, drawing on data from the 2019 UK CF registry report.^19^ Health states 3 through 6 considered variants of the base case reflecting the following: no PEx requiring IV treatment (health state 3), three PExs requiring IV treatment per year (health state 4), an additional nebulized medicine (health state 5), or an additional physiotherapy session (health state 6). Following the guidance of patient experts, location of IV antibiotic administration was not specified in the vignettes. The comparator health state for all TTO exercises (labeled best possible health) used the EQ-5D-5L descriptive system, with additional text stating that this health state was the following: no CF or any other preexisting conditions and no medications.

### TTO Procedure

**Figure 2:**
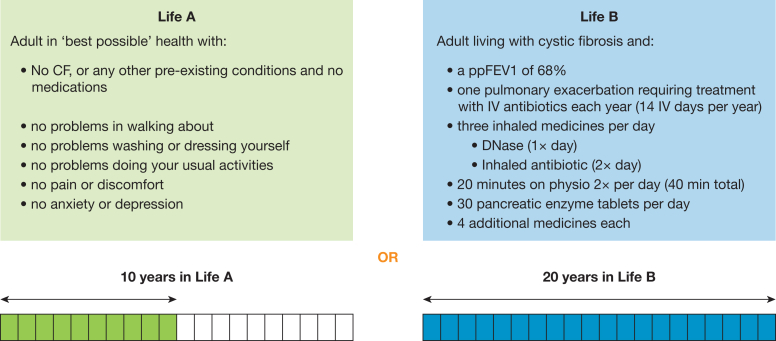
An example time trade-off task for the valuation of health state 2. CF = cystic fibrosis; DNase = Deoxyribonuclease; ppFEV1= FEV_1_ % predicted.

Interviews were conducted between November 2020 and March 2021. Two interviewers (D. O. and J. M.) received formal interview training prior to study fieldwork. Interviews were administered by video call using a semistructured interview guide. An equal number of interviews was conducted by the interviewers. A detailed overview of the interview flow is shown in e-Appendix 1. The interview schedule was based on the EQ-VT v2 TTO protocol for valuation of health states described by the EQ-5D-5L HRQoL measure.^20^ Prior to the TTO tasks, participants were asked to rate their current health using the EQ-5D-5L and EuroQol visual analog scale. Interviewers then walked participants through the TTO task using the health state example of living in a wheelchair; this example was also used to demonstrate how health states could be valued as worse than being dead. Participants were then given a practice task using a health state described by the EQ-5D-5L system. The health states to be valued were then introduced and participants were asked to rank the states in order of preference, and whether they considered the states to be better or worse than being dead. In the main trade-off exercise, participants were first asked to value their own current health, followed by the base case, with subsequent tasks presented in random order. An example TTO task is shown in Figure 2.

Based on patient expert feedback during study design, the time horizon for living in the state being valued was extended from the more common 10 to 20 years. For health states considered better than dead, choices were presented in 2-year increments, with the final choice using a 1-year increment. At the point at which the respondent indicated indifference between *x* years in the best possible health state and 20 years in the alternative, HSUs were calculated as the ratio of the two choices (*x*/20). Although no respondent rated any health state as worse than death, the facility to do so was enabled in the interview schedule, with an iteration procedure as per the EQ-VT protocol, using a 10-year horizon.^20^

Additional questions asked during the interview included the EQ-5D-5L HRQoL scale and EuroQol visual analog scale; the Cystic Fibrosis Quality of Life Questionnaire (CFQoL) treatment issues domain; and a feedback section that included a chance to view and comment on the ratings given for the health states, a 7-point Likert scale assessing the difficulty of the task, and open-ended questions on the tasks and participants’ experience of treatment burden. Responses to the Likert scale were trichotomized into difficult (points 1-3), neither difficult nor easy (point 4), and easy (points 5-7). Responses to the open-ended questions were grouped into themes.

### Statistical Analysis

Inconsistency in response to the TTO exercise was defined as valuing an objectively inferior health state higher than an objectively superior one (eg, health state 4 > health state 3). Because no generalizable rule exists for the treatment of inconsistent responses,^21^ the primary analysis included all responses on the basis that representativeness may be effected if particular subgroups are more likely to respond inconsistently than others.^22^ Differences in responses to the feedback questions at the end of the interview between consistent and inconsistent responders were assessed using χ^2^ tests.

Descriptive statistics were used to describe the sample and crude utility values. All pairwise comparison of mean utility values (between different HSUs and subgroup analyses) were assessed using paired *t* tests. The EQ-5D-5L measure was valued using the UK cross-walk algorithm.^23^ Agreement between the EQ-5D-5L and TTO instruments for valuing participants’ own health was assessed using the Lin concordance correlation coefficient and visualized with Bland-Altman plots.

Mixed effects linear regressions were used to model the utility decrement of additional treatment occasions (IV antibiotics, inhaled medicines, or physiotherapy). A respondent-level random intercept was used to account for multiple observations per respondent; all other parameters were treated as fixed. Additional inhaled medicine or physiotherapy treatment occasions were specified as dummy variables. PEx requiring IV antibiotics was specified as linear after using Wald tests to verify a linear relationship with utility. During model selection, respondent characteristics hypothesized to impact TTO responses (demographics, clinical measures, EQ-5D-5L index value, EuroQol visual analog scale) were included in adjusted models, with parameter inclusion guided by improvement in Akaike information criteria across models. The final model included age, sex, and FEV_1_ % predicted (ppFEV_1_). All analyses were conducted using Stata IC (StataCorp).

## Results

### Participant Characteristics

Of the 52 participants, 51 completed the interviews, with one patient dropping out due to technical problems with the video link.

The sample (Table 1) was 53% female, with a median age of 30 years (range, 19-66) and mean ppFEV_1_ function of 65% (SD, 20%). The sample was similar to the center’s CF population regarding age (median, 33.6 years) and lung function (mean ppFEV_1_, 68).^24^ Participants spent an average of > 2 hours on treatment each day, and almost one-half had required IV treatment for PEx in the previous year. The mean EuroQol visual analog scale score was 75 (SD, 12), whereas the mean EQ-5D index value was 0.81 (SD, 0.20), within the range of previously reported HSU values for pwCF.^11^^,^^25^ Participants indicated a relatively high level of perceived treatment burden, with a mean CFQoL score of 58 (SD, 26).

**Table 1:** Characteristics of Study Participants

Characteristic	No. of Patients	Mean (SD) or No. (%)	Median (IQR)
Age, y	51	33 (11)	30 (14)
Sex, female	51	27 (53%)	NA
ppFEV_1_, L	51	65 (20)	69 (31)
Mild, > 70%		24 (47%)	NA
Moderate, 40%-70%		21 (41%)	NA
Severe, < 40%		6 (12%)	NA
Required IV antibiotics in last 12 mo	47	23 (49%)	NA
Prescribed a CFTR modulator	51	36 (71%)	NA
Elexacaftor/tezacaftor/ivacaftor		28 (55%)	NA
Tezacaftor/ivacaftor		5 (10%)	NA
Ivacaftor		3 (6%)	NA
Total treatment time, min/d	51	125 (137)	96 (80)
EQ-5D index value^a^	51	0.81 (0.20)	0.86 (0.16)
EQ visual analog scale score^b^	51	75 (12)	75 (15)
CFQoL treatment issues domain score^c^	47	58 (26)	57 (47)

CFQoL = Cystic Fibrosis Quality of Life Questionnaire; CFTR = cystic fibrosis transmembrane conductance regulator; IQR = interquartile range; NA = not applicable; ppFEV_1_ = FEV_1_ % predicted.

a EQ-5D Index is scored from 0 to 1, where 1 is full health and 0 is dead.

b EQ-5D visual analog scale is scored on a scale of 0 to 100, where 0 is worst possible health and 100 is best possible health.

c CFQoL is scored on a scale of 0 to 100, where 0 is very high level of treatment issues and 100 is no treatment issues.

### TTO Results

#### Unadjusted Mean HSU Estimates

The crude unadjusted mean HSUs are shown in Table 2. The mean HSU value for participants’ current health was 0.82, compared with 0.81 as valued by the EQ-5D-5L. Despite similar mean values, there appeared to be limited agreement between the two instruments for individual participants (Lin concordance correlation coefficient = 0.33), particularly at lower HSU values (e-Fig 1). No statistically significant associations with current HSU were observed for any participant characteristics measured in this study. No participant perceived any of the health states evaluated to be worse than dead, and there were therefore no negative HSU values.

**Table 2:** Crude Unadjusted Mean Health State Utilities (N = 51)

Health State	Mean (SD)	95% CI	*P* Value^a^
Current health, health state 1	0.82 (0.20)	0.76-0.87	.80
Base case, health state 2	0.80 (0.18)	0.75-0.85	NA
Base case with no PEx requiring IV abx, health state 3	0.84 (0.16)	0.80-0.86	.003
Base case with three PEx requiring IV abx, health state 4	0.73 (0.23)	0.66-0.80	.0001
Base case with additional nebulized medicine, health state 5	0.78 (0.20)	0.73-0.84	.30
Base case with additional physiotherapy, health state 6	0.77 (0.22)	0.71-0.83	.10

abx = antibiotics; NA = not applicable; PEx = pulmonary exacerbation.

a Paired *t* tests: health state 1 vs EQ-5D index; health state 3-6 vs health state 2.

#### Analysis of Response Consistency

Seventeen participants (33%) gave inconsistent responses in the TTO vignette tasks (e-Table 1). No statistically significant differences were observed between participants with consistent responses and those with inconsistent responses in terms of demographic, clinical, or HRQoL parameters (e-Table 2).

Crude mean HSUs for the TTO vignettes decreased or increased in a logically consistent manner with corresponding changes in the health states described. Nominal reductions in HSU for additional physiotherapy or additional inhaled medicine were not statistically significant when all participant data were included; however, significant differences were observed when only consistent responders were included in the analysis (e-Table 3).

Although only two respondents did not trade life years in the exercises (ie, HSU for all states = 1), a further nine respondents (18%) traded an equal number of years for all health states, indicating they saw no discernible differences between the vignettes in terms of HSU. This equal trading group tended to be older than those who did indicate differences (mean age, 41 vs 32 years; *P* = .02), but no other clinical or demographic differences were observed between the two groups.

#### Respondent Feedback

Just under one-half of the participants indicated they found the tasks slightly or moderately difficult (e-Table 4); however, a greater proportion of inconsistent responders found the tasks difficult (71% vs 35%, *P* = .06) (e-Table 4). Similarly, inconsistent responders were more likely to indicate they would give different answers if they were to repeat the task (41% vs 12%, *P* = .02) (e-Table 5).

In open-ended feedback (Table 3), only one respondent suggested that changes between health states were insufficient to warrant trading life years. Meanwhile, almost 20% of the sample indicated nonhealth factors influenced their decision, including their age and having someone to live for.

**Table 3:** Themes From Open-Ended Feedback From Participants

Theme	No. Making Comments in This Theme	Example Quotes
Having someone to live for influenced trading decisions	7 (of these, 4 rated all health states with the same value)	“I’ve had a lot of time to think about this. As a parent my life isn’t all my own, responsibilities can’t be ignored.” “I will always choose to maximize my years of life, no matter how hard it gets so I can stay with my family.”
Older participant age influenced trading decisions	3 (all rated all health states with the same value)	“I am retired now so treatment burden doesn’t have such a big impact—answers may be different if I was still working as I had to have more routine and it was a lot harder.” “As I get older it’s something I think about more [as I have] less years to live.”
Differences between health states in vignettes not big enough to justify trading life years	1	“I didn’t see a big enough change. . . which would encourage me to trade off any time”

#### Estimation of Utility Decrements Associated With Additional Treatment Occasions

The utility decrements associated with additional PEx, additional physiotherapy, and additional inhaled medicine use, as estimated by the mixed effects regression model, are presented in Table 4 (point estimates for the utility impact of changes in PEx associated with health states 3 and 4 are provided in e-Table 6).

**Table 4:** Utility Decrement Estimates

Parameter	Parameter Estimate	SE	95% CI
Health state 3 and 4: additional PEx requiring IV abx,^a^ per year	−0.037^b^	0.008	−0.052 to −0.022
Health state 5: additional nebulized medicine, additional 25 min/d	−0.019	0.013	−0.044 to 0.006
Health state 6: additional 20-min physiotherapy session, per day	−0.029^c^	0.014	−0.055 to −0.002
Male	−0.031	0.053	−0.13 to 0.072
Age^d^	−0.002	0.003	−0.008 to 0.003
ppFEV_1_^d^	0.0003	0.001	−0.002 to 0.002
Intercept	0.85^b^	0.032	0.79 to 0.92

abx = antibiotics; PEx = pulmonary exacerbation; ppFEV_1_ = FEV_1_ % predicted.

a PEx was specified as having a linear relationship with utility: this parameter estimate should be interpreted as the utility decrement associated with each additional PEx event.

b *P* < .001.

c *P* < .05.

d Parameters are centered on the mean (age, 33 y; ppFEV_1_, 65%).

The largest decrement was observed for additional PEx requiring IV antibiotics (utility decrement, −0.037; *P* < .001), followed by additional physiotherapy (utility decrement, −0.029; *P* < .05) and additional inhaled medicine use (utility decrement, −0.019; *P* < .10). Estimates of a similar magnitude were observed when the model was restricted to consistent responders only (e-Table 7).

## Discussion

The objective of this study was to estimate the utility loss associated with additional treatment occasions, ultimately to facilitate the consideration of changes in treatment burden in the economic evaluation of novel interventions for the management of CF. For all aspects of treatment burden investigated, most participants indicated a preference to avoid additional treatment burden by trading life years. Increasing treatment burden was associated with decreasing crude HSU values for each aspect, with the greatest effect observed for changes in the frequency of IV antibiotic treatments of PEx. Mixed regression models showed statistically meaningful utility decrements associated with physiotherapy and IV antibiotic treatment of PEx. Minimally important differences reported for utility scores vary considerably between instruments, but typically range from 0.03 to 0.06.^12^^,^^26^ Disutility estimates in this study straddle the lower end of that range, but nonetheless may suggest they represent meaningful differences in HRQoL for patients with CF.

Our findings are in general agreement with the limited existing literature on the HRQoL impact of treatment burden. A vignette-based TTO study investigating treatment preferences for different modes of inhaled treatment administration reported an HSU decrement of 0.09 from using a nebulized inhaler rather than a dry powder inhaler.^27^ Although this is a greater decrement than we found (and our decrement did not reach significance at the 5% level), our study explored the utility impact of an additional inhaled medication, rather than of a different mode of administration. Utility estimates from a study by Gold et al^28^ using the EQ-5D-5L among pwCF receiving inpatient IV antibiotic treatment for PEx suggest a mean HSU improvement of approximately 0.1 from onset of PEx to 28 days later. This would equate to a considerably smaller estimate quality-adjusted life years impact than our estimate (calculated as follows: HSU × [duration of health state/1 year]).

One-fifth of the participants rated all health states with the same value (including two nontraders). Although this observation may suggest that a sizeable proportion of the sample saw no discernible difference between the health states evaluated, only one respondent indicated such in the postexercise feedback. It is noteworthy that these 11 respondents tended to be older. Although evidence regarding the impact of age on TTO valuations is mixed,^29^ “having someone to live for” has been shown to reduce the extent to which TTO participants are willing to trade life years.^30^ A similar finding was reflected in the feedback from participants in our study: of the 11 respondents rating all health states the same, four indicated family commitments had a significant bearing on their answers, whereas three indicated that growing older reduced the relative importance of treatment burden.

Participants’ valuation of their own health using TTO produced similar crude mean utility values to those calculated using the EQ-5D-5L measure, and were comparable to values described for CF populations in the published literature.^11^^,^^25^ However, the limited concordance observed between the EQ-5D-5L index value and the TTO valuation of own health may suggest the two instruments consider different constructs. Alternatively, it may be that similar constructs are being considered, but the different populations canvassed in valuation studies (general public for EQ-5D-5L; patients in this study) place different values on them.

### Strengths and Limitations

An interviewer-facilitated online approach enabled preference elicitation in a manner that avoided known issues with engagement and comprehension of the TTO task in online studies.^31^ Because this study was conducted during the COVID-19 pandemic, this approach was a safe, pragmatic alternative to in-person interviewing of a vulnerable population. Inclusion of generic and disease-specific patient-reported outcomes enabled broader understanding of HRQoL in the sample population, and the relative impact of treatment burden.

Our results must be considered in the context of several limitations. First, designed as a feasibility study, the relatively small sample from a single center may limit the generalizability of the study findings.

Focusing only on treatment burden-related attributes may introduce a degree of framing bias; however, expanding attributes to be tested in the vignettes would have necessitated increased sample size and interviewer time beyond the resources available to the study.

Preferences for health are known to be influenced by respondent characteristics.^29^ Although no association was found between recorded respondent characteristics and reported HSUs, unobserved characteristics may have influenced participants’ willingness to trade (eg, having someone to live for).

Inconsistent responses reduced the precision of the estimated HSUs. Unfortunately, inconsistent responses are a known issue in TTO methodology.^21^^,^^32^ The level of inconsistency in this study (33%) is within the range of levels found in large-scale valuation studies for the EQ-5D (12%-79%).^22^^,^^31^^,^^33^ Because people tend to learn during the TTO tasks, adding additional practice tasks may have helped reduce levels of inconsistency.^34^ Because inconsistencies are most likely to result in attenuation of the differences in values between HSUs, this may lead to an underestimation of health benefits when deployed in economic evaluations.^22^

Limitations related to the TTO methodology itself have been widely discussed: it is subject to confounding by respondents’ time preferences and life expectancies,^30^ interviewer effects,^35^ and cognitive understanding.^35^ In this study, several participants indicated factors beyond just the health states that influenced their decision. A small number indicated that the health states shown were quite different from their own experience of CF and were therefore quite difficult to conceptualize. This is a shortcoming associated also (and potentially more strongly) with health state valuation by the general public.^12^

The extent to which HSUs elicited from pwCF in this study diverge from those of the general population is not known. Patient valuations commonly lead to higher utility weightings than the general population, explained by both adaptation to the health state and response shifts in values that result from changing circumstances or experiences.^36^

Despite these limitations, the general agreement of estimates of respondents’ current health, and the base case adult with CF health state with estimates from the literature,^11^^,^^37^ supports the validity of our approach.

## Interpretation

This study highlights the potential for undervaluation of patient benefits when using generic HRQoL measures. It suggests treatment burden is an important outcome to pwCF that should be considered as a secondary outcome in trials of novel CF interventions. In the absence of preference-based tools incorporating a treatment burden dimension, a potential use of these utility value data may be as sensitivity analyses when conducting economic evaluations of interventions anticipated to have beneficial impacts on PEx or treatment burden.^38^

## Funding/Support

This study was funded by an 10.13039/501100000272NIHR Research for Patient Benefit Grant [Grant PB-PG-1217-20018]. J. A. W. and R. A. C. were also supported by the National Institute of Health Research (NIHR) Applied Research Collaboration East of England program. Views expressed are those of the authors and not necessarily those of the NHS, the NIHR, or the Department of Health.

## Financial/Nonfinancial Disclosures

The authors have reported to *CHEST Pulmonary* the following: N. J. S. has received honoraria for lectures from Vertex, Gilead, Chiesi, Teva, and Zambon; and fees for advisory boards from Vertex, Gilead, Chiesi, and Menarini. S. B. C. has received honoraria for lectures from Vertex and Chiesi; and fees for advisory boards or consulting from Vertex, Chiesi, and Profile Pharma. None declared (R. A. C., J. M., D. O., M. R., J. A., J. A. W.).

## Supplementary Data

e-Online Data

## References

[1] Bell S.C., Mall M.A., Gutierrez H. (2020). The future of cystic fibrosis care: a global perspective. Lancet Respir Med.

[2] Carnovale V., Iacotucci P., Terlizzi V. (2022). Elexacaftor/tezacaftor/ivacaftor in patients with cystic fibrosis homozygous for the F508del mutation and advanced lung disease: a 48-week observational study. J Clin Med.

[3] McCoy K.S., Blind J., Johnson T. (2023). Clinical change 2 years from start of elexacaftor-tezacaftor-ivacaftor in severe cystic fibrosis. Pediatr Pulmonol.

[4] Desai M., Hine C., Whitehouse J.L., Brownlee K., Charman S.C., Nagakumar P. (2022). Who are the 10%? - non eligibility of cystic fibrosis (CF) patients for highly effective modulator therapies. Respir Med.

[5] Rowbotham N.J., Smith S., Elliott Z.C. (2023). A refresh of the top 10 research priorities in cystic fibrosis. Thorax.

[6] Smyth A.R., Bell S.C., Bojcin S. (2014). European Cystic Fibrosis Society standards of care: best practice guidelines. J Cyst Fibros.

[7] Konstan M.W., Flume P.A., Kappler M. (2011). Safety, efficacy and convenience of tobramycin inhalation powder in cystic fibrosis patients: The EAGER trial. J Cyst Fibros.

[8] Wildman M.J., O’Cathain A., Maguire C. (2022). Self-management intervention to reduce pulmonary exacerbations by supporting treatment adherence in adults with cystic fibrosis: a randomised controlled trial. Thorax.

[9] Mayer-Hamblett N., Ratjen F., Russell R. (2022). Discontinuation versus continuation of hypertonic saline or dornase alfa in modulator treated people with cystic fibrosis (SIMPLIFY): results from two parallel, multicentre, open-label, randomised, controlled, non-inferiority trials. Lancet Respir Med.

[10] Acaster S., Mukuria C., Rowen D. (2023). Development of the Cystic Fibrosis Questionnaire-Revised-8 dimensions: estimating utilities from the Cystic Fibrosis Questionnaire-Revised. Value Health.

[11] Acaster S., Pinder B., Mukuria C., Copans A. (2015). Mapping the EQ-5D index from the cystic fibrosis questionnaire-revised using multiple modelling approaches. Health Qual Life Outcomes.

[12] Brazier J., Ratcliffe J., Saloman J., Tsuchiya A. (2017).

[13] Drummond M.F., Sculpher M.J., Claxton K., Stoddart G.L., Torrance G.W. (2015).

[14] Carr S., Archangelidi O., Keogh K., Abbott J., Whitty J.A., Simmonds N.J. (2019). Evidence-based valuation of patient-centred outcomes in Cystic Fibrosis. National Institute for Health Research (NIHR).

[15] Dolan P., Gudex C., Kind P., Williams A. (1996). The time trade-off method: results from a general population study. Health Econ.

[16] Matza L.S., Stewart K.D., Lloyd A.J., Rowen D., Brazier J.E. (2021). Vignette-based utilities: usefulness, limitations, and methodological recommendations. Value Health.

[17] Cameron R.A., Office D., Matthews J. (2022). Treatment preference among people with cystic fibrosis: the importance of reducing treatment burden. Chest.

[18] Altabee R, Carr S, Abbott J (2024). Evaluating the correspondence between the EQ-5D-5L and disease severity and quality of life in adults and adolescents with cystic fibrosis. Respir Med Res.

[19] Charman S., Lee A., Cosgriff R., McClenaghan E., Carr S. (2020).

[20] Stolk E., Ludwig K., Rand K., van Hout B., Ramos-Goni J.M. (2019). Overview, update, and lessons learned from the international EQ-5D-5L valuation work: version 2 of the EQ-5D-5L valuation protocol. Value Health.

[21] Devlin N.J., Hansen P., Kind P., Williams A. (2003). Logical inconsistencies in survey respondents' health state valuations - a methodological challenge for estimating social tariffs. Health Econ.

[22] Yang Z., van Busschbach J., Timman R., Janssen M.F., Luo N. (2017). Logical inconsistencies in time trade-off valuation of EQ-5D-5L health states: Whose fault is it?. PLoS One.

[23] van Hout B., Janssen M.F., Feng Y.S. (2012). Interim scoring for the EQ-5D-5L: mapping the EQ-5D-5L to EQ-5D-3L value sets. Value Health.

[24] Shaikh N., Lee A., Charman S., Cosgriff R., Carr S. (2021).

[25] Chevreul K., Michel M., Brigham K.B. (2016). Social/economic costs and health-related quality of life in patients with cystic fibrosis in Europe. Eur J Health Econ.

[26] McClure N.S., Sayah F.A., Xie F., Luo N., Johnson J.A. (2017). Instrument-defined estimates of the minimally important difference for EQ-5D-5L Index scores. Value Health.

[27] Sawicki G.S., Goldman D.P., Chan W., Casey A., Greenberg J., Romley J.A. (2015). Patient preference for treatment administration in cystic fibrosis. Am J Pharm Benefits.

[28] Gold L.S., Patrick D.L., Hansen R.N., Beckett V., Goss C.H., Kessler L. (2019). Correspondence between symptoms and preference-based health status measures in the STOP study. J Cyst Fibros.

[29] van Nooten F., Busschbach J., van Agthoven M., van Exel J., Brouwer W. (2018). What should we know about the person behind a TTO?. Eur J Health Econ.

[30] Hansen T.M., Stavem K., Rand K. (2022). Time trade-off with someone to live for: impact of having significant others on time trade-off valuations of hypothetical health states. Qual Life Res.

[31] Jiang R., Shaw J., Muhlbacher A. (2021). Comparison of online and face-to-face valuation of the EQ-5D-5L using composite time trade-off. Qual Life Res.

[32] Badia X., Roset M., Herdman M. (1999). Inconsistent responses in three preference-elicitation methods for health states. Soc Sci Med.

[33] Lamers L.M., Stalmeier P.F.M., Krabbe P.F.M., Busschbach J.J.V. (2006). Inconsistencies in TTO and VAS values for EQ-5D health states. Med Decis Making.

[34] Attema A.E., Edelaar-Peeters Y., Versteegh M.M., Stolk E.A. (2013). Time trade-off: one methodology, different methods. Eur J Health Econ.

[35] Lugnér A.K., Krabbe P.F.M. (2020). An overview of the time trade-off method: concept, foundation, and the evaluation of distorting factors in putting a value on health. Expert Rev Pharmacoecon Outcomes Res.

[36] Brazier J., Akehurst R., Brennan A. (2005). Should patients have a greater role in valuing health states?. Appl Health Econ Health Policy.

[37] Mohindru B., Turner D., Sach T. (2020). Health state utility data in cystic fibrosis: a systematic review. Pharmacoecon Open.

[38] Brazier J., Ara R., Azzabi I. (2019). Identification, review, and use of health state utilities in cost-effectiveness models: An ISPOR Good Practices for Outcomes Research Task Force Report. Value Health.

