# Supplementary material for: Health State Utilities Associated With Treatment Burden in Cystic Fibrosis: A Patient Valuation Study
Source: CHEST Pulm. 2024 Aug 23;3(1):100097. doi: 10.1016/j.chpulm.2024.100097 (PMC13419222; doi:10.1016/j.chpulm.2024.100097)

- ***Rev 06.TTO INTERVIEW FLOW***

1. GENERAL WELCOME
   - Objectives of project & interview
   - Hypothetical nature of questions
   - How data will be used
   - Right to withdraw at any time
   - Informed consent
2. EUROQOL EQ-5D-5L QUESTIONNAIRE
   - Self-reported health on the EQ-5D-5L
   - Self-reported health on the EQ-VAS
3. TIME TRADE-OFF
   - Instructions and examples of TTO task
     1. Interviewer demonstrates living in a wheelchair TTO exercise
     2. Interviewer repeats living in a wheelchair TTO exercise to introduce valuation of states worse than death
   - Practice/ warm up exercise
     1. EQ-5D-5L health state 15411 valued (against EQ-5D-5L 11111)
   - Main TTO exercises
     1. 6 health states introduced
     2. Participant rank orders all six health states according to preference
     3. TTO 1 participant values own current health against EQ-5D-5L 11111
     4. TTO 2 participant values base case (HS2) against EQ-5D-5L 11111
     5. TTO 3-6 random order valuation against EQ-5D-5L 11111 of
        1. HS3 (no exacerbations)
        2. HS4 (3x exacerbations/year)
        3. HS5 (4x inhaled medications)
        4. HS6 (3x physio sessions)
     6. Participant shown values given for all six health states & asked if they agree with these scores, or if they would make changes
4. WRAP-UP
   - Semi- structured feedback
     1. 7-point Likert scale “How difficult did you find the trade-off tasks?”
     2. Open-ended questions:
        - What was difficult about the task?
        - What do you consider to be treatment burden (or how would you define treatment burden)?
        - What element of your treatment do you struggle with the most?
        - What other aspects of your treatment do you feel have a big impact on your quality of life?
        - On a typical day, how much time (in minutes) would you say you spend on:
          - Physio
          - Inhaled medicines
          - Other aspects of treatment
        - Is there any further feedback on the tasks that you would like to add?

**eTable 1:** Response to question “would you make changes to these scores if you were to repeat the exercises?”

|  | All (n=51)  No. (%) | Consistent responders (n=34)  No. (%) | Inconsistent responders (n=17)  No. (%) |
| --- | --- | --- | --- |
| Would not change scores | 40 (78) | 30 (88) | 10 (59) |
| Would change scores | 11 (22) | 4 (12) | 7 (41) |
| p=0.02 (Χ^2^ test) | | | |

**eTable 2:** Ease of completion of the TTO tasks

|  | All (n=51)  No. (%) | Consistent responders (n=34)  No. (%) | Inconsistent responders (n=17)  No. (%) |
| --- | --- | --- | --- |
| Extremely easy | 7 (14) | 6 (18) | 1 (6) |
| Moderately easy | 11 (22) | 9 (26) | 2 (12) |
| Slightly easy | 3 (6) | 2 (6) | 1 (6) |
| Total easy | **21 (41)** | **17 (50)** | **4 (24)** |
| Neither difficult nor easy | **6 (12)** | **5 (15)** | **1 (6)** |
| Slightly difficult | 14 (27) | 7 (21) | 7 (41) |
| Moderately difficult | 10 (20) | 5 (15) | 5 (29) |
| Extremely difficult | 0 (0) | 0 (0) | 0 (0) |
| Total difficult | **24 (47)** | **12 (35)** | **12 (71)** |
| p=0.06 (Χ^2^ test for three-category table [Total easy, Neither difficult nor easy, Total difficult]) | | | |

**eTable 3:** Summary of inconsistent responses

|  | Inconsistent responders (n=17)  No. (%) |
| --- | --- |
| Number of inconsistent responses  1  2  3 | 9 (53)  6 (35)  2 (12) |
| Base case with no exacerbations requiring IV abx (HS3) | 5 (29) |
| Base case with 3 exacerbations requiring IV abx (HS4) | 5 (29) |
| Base case with additional nebulised medication (HS5) | 9 (53) |
| Base case with additional physiotherapy (HS6) | 8 (47) |

**eTable 4:** Comparison of characteristics of consistent responders and inconsistent responders

| Characteristics (n=51) | Sample number  (consistent, inconsistent) | Consistent responses  Mean (SD) or No. ( %) | Inconsistent responses  Mean (SD) or No. ( %) | p  (t-test or Χ^2^) |
| --- | --- | --- | --- | --- |
| Age (years) | 34, 17 | 34 (12) | 32 (9) | 0.6 |
| Sex (female) | 34, 17 | 16 (47%) | 11 (65%) | 0.2 |
| ppFEV1 (l)  Mild (>70%)  Moderate (40-70%)  Severe (<40%) | 34, 17 | 65 (21)  14 (41%)  16 (47%)  4 (12%) | 67 (20)  10 (59%)  5 (29%)  2 (12%) | 0.7  0.4 |
| Required IV antibiotics in last 12 months | 31, 16 | 17 (55%) | 6 (38%) | 0.3 |
| Prescribed a CFTR modulator  elexacaftor/tezacaftor/ivacaftor | 34, 17 | 24 (71%)  20 (59%) | 11 (65%)  47 (47%) | 0.7  0.6 |
| Total treatment time (mins/day) | 33, 17 | 126 (95) | 125 (198) | 0.99 |
| EQ-5D Index score | 34, 17 | 0.81 (22) | 0.82 (16) | 0.8 |
| EQ-5D VAS score | 34, 17 | 75 (14) | 76 (10) | 0.8 |
| CFQoL treatment burden domain score | 31, 16 | 56 (26) | 62 (27) | 0.4 |

**eTable 5:** Crude health state utilities for consistent responders

| Health state (n=34) | Mean | 95% CI | p^†^ |
| --- | --- | --- | --- |
| Current health (HS1) | 0.85 | 0.79, 0.91 | 0.3 |
| Base case (HS2) | 0.84 | 0.79, 0.9 | - |
| Base case with no exacerbations requiring IV abx (HS3) | 0.88 | 0.83, 0.92 | 0.003 |
| Base case with 3 exacerbations requiring IV abx (HS4) | 0.76 | 0.68, 0.84 | 0.0003 |
| Base case with additional nebulised medication (HS5) | 0.82 | 0.76, 0.88 | 0.02 |
| Base case with additional physiotherapy (HS6) | 0.81 | 0.74, 0.88 | 0.02 |
| † paired t-tests: HS1 *vs* EQ-5D Index; HS3-6 *vs* HS2 | | | |

**eTable 6:** Utility decrement estimates, with point estimates for PEx changes in HS3 & HS4

| Parameter | Parameter estimate | SE | 95% CI |
| --- | --- | --- | --- |
| HS3: No PEx requiring IV abx  (per year) | 0.044** | 0.014 | 0.016, 0.072 |
| HS4: Three additional PEx requiring IV abx  (per year) | -0.070*** | 0.016 | -0.10, -0.038 |
| HS5: Additional nebulised medicine  (additional 25 mins/ per day) | -0.015 | 0.015 | -0.044, 0.013 |
| HS6: Additional 20 minute physiotherapy session  (per day) | -0.025* | 0.015 | -0.054, 0.005 |
|  |  |  |  |
| Male | -0.031 | 0.053 | -0.13, 0.072 |
| Age^†^ | -0.002 | 0.003 | -0.008, 0.003 |
| ppFEV1^†^ | 0.0002 | 0.001 | -0.002, 0.002 |
| Intercept | 0.81*** | 0.035 | 0.74, 0.88 |
| * p <0.1 ** p<0.05 *** p<0.001  ^†^ Parameters are centred on the mean (age, 33 years; ppFEV1, 65%) | | | |

**eTable 7:** Utility decrement estimates for consistent responders

| Parameter | Parameter estimate | SE | 95% CI |
| --- | --- | --- | --- |
| HS3 & HS4: Additional PEx requiring IV abx^†^  (per year) | -0.039*** | 0.009 | -0.057, -0.020 |
| HS5: Additional nebulised medicine  (additional 25 mins/ per day) | -0.018* | 0.009 | -0.036, 0.0001 |
| HS6: Additional 20 minute physiotherapy session (per day) | -0.028 | 0.017 | -0.061, 0.005 |
|  |  |  |  |
| Male | -0.088* | 0.047 | -0.19, 0.01 |
| Age^††^ | -0.002 | 0.003 | -0.008, 0.003 |
| ppFEV1^††^ | 0.001 | 0.001 | -0.001, 0.004 |
| Intercept | 0.93*** | 0.02 | 0.88, 0.97 |
| * p <0.1 ** p<0.05 *** p<0.001  ^†^PEx was specified as having a linear relationship with utility: this parameter estimate should be interpreted as the utility decrement associated with each additional PEx event  ^††^Parameters are centred on the mean (age, 33 years; ppFEV1, 65%) | | | |

**eFigure 1:** Bland Altman plot showing agreement between health state utility scores for the TTO and EQ-5D-5L instruments


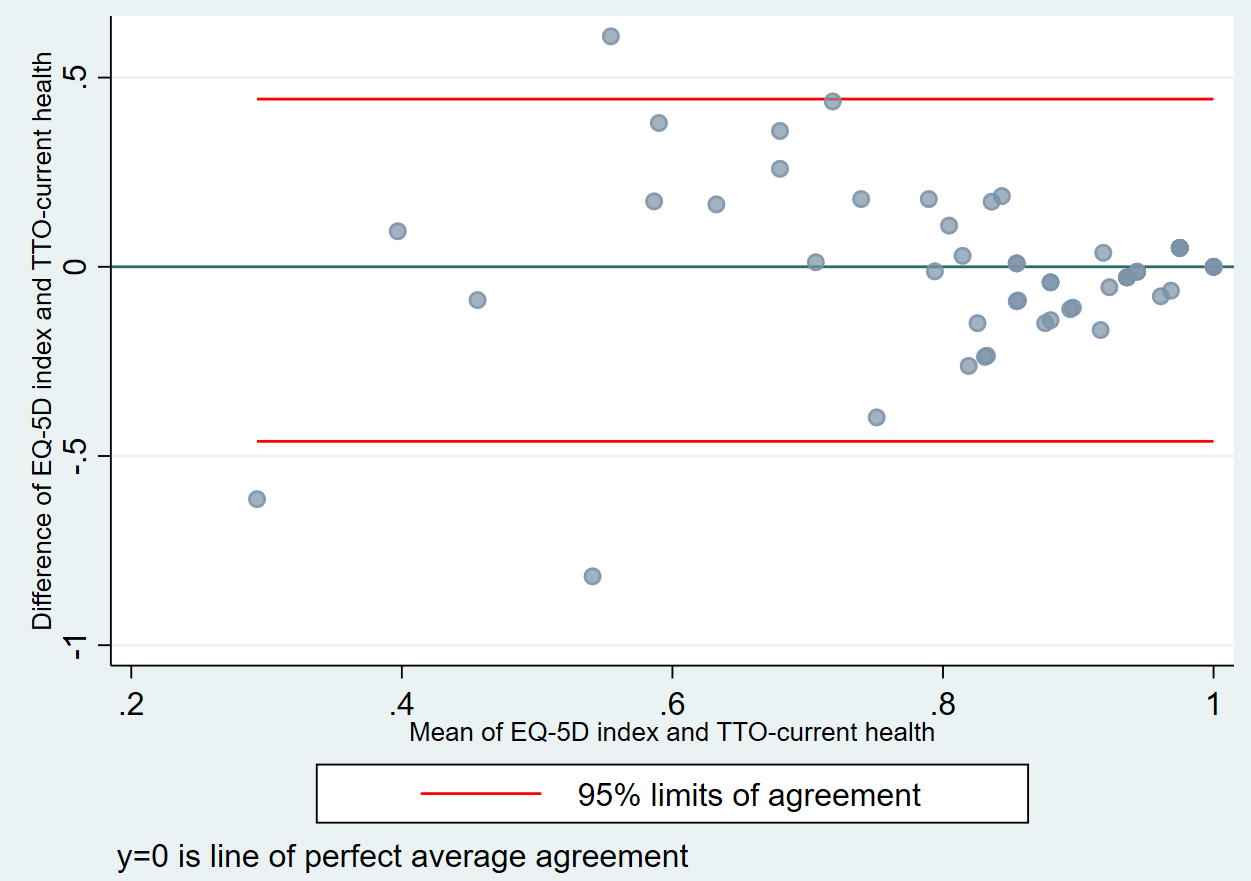

Supplement: e-Online Data [file mmc1.docx]
